# Fabric muscle with a cooling acceleration structure for upper limb assistance soft exosuits

**DOI:** 10.1038/s41598-022-15682-w

**Published:** 2022-07-06

**Authors:** Seong Jun Park, Kyungjun Choi, Hugo Rodrigue, Cheol Hoon Park

**Affiliations:** 1 Department of Robotics and Mechatronics, Korea Institute of Machinery and Materials, Daejeon, 34103 Korea; 2 School of Mechanical Engineering, Sungkyunkwan University, Suwon, 16419 Korea; 3 Department of Mechatronics Engineering, Chungnam National University, Daejeon, 34134 Korea

**Keywords:** Engineering, Materials science

## Abstract

Soft exosuits used for supporting human muscle strength must be lightweight and wearable. Shape memory alloy (SMA) spring-based fabric muscles (SFM) are light and flexible, making them suitable for soft and shape-conformable exosuits. However, SFMs have a slow actuation speed owing to the slow cooling rate of the SMA spring. This paper proposes a forced air-cooling fan-integrated fabric muscle (FCFM) that improves the cooling rate by arranging a thin-diameter SMA spring bundle with a high surface-area-to-volume ratio inside a breathable fabric with integrated fans. The relaxation time of an FCFM weighing 30 g and containing a 2.6 g SMA spring bundle, which contains 200 thin springs, was reduced by over 70.2% via forced-air cooling using the integrated fans. A 4 kg weight, which is 1530 times the mass of the SMA spring bundle, was hung from the FCFM and was repeatedly actuated in ten-second cycles. An upper limb assistive soft exosuit with FCFMs was fabricated and worn on a mannequin holding a dumbbell, and the arm extension time after flexion was improved by 4.5 times. Additionally, the assistive performance of the exosuits for repetitive tasks in specific scenarios was evaluated, and the strong potential of the proposed FCFM for soft exosuits was verified.

## Introduction

Wearable robots can assist humans in performing tasks by supporting human muscle strength^1–4^. Light and soft materials are used in soft wearable robots (exosuits) to easily fit the wearer’s body shape and realize free and comfortable motion while minimizing the increase in mass or inertia. Exosuits often utilize tendon-driven mechanisms using cable-driven actuators^5–8^ or twisted string actuators^9–12^. However, the required driving and routing mechanisms increase the complexity, weight, and volume of the system. Exosuits using pneumatic actuators^13–16^ are light and soft. However, they are complex, noisy, heavy, and bulky owing to the additional components required for generating and controlling pneumatic pressure.

Skeletal muscles are evenly distributed around the bones. Researchers have been studying fabric muscles, i.e., fabric-type soft actuators that can be directly and evenly distributed in the area to be assisted similarly to skeletal muscles. Light and flexible materials such as twisted and coiled polymer actuators (TCPA) and shape memory alloy (SMA) actuators have been used in fabric muscles^17–23^. These actuators are contracted and actuated via Joule heating. TCPA, which has a slow cooling rate, must be heated to temperatures exceeding 100 °C for sufficient contraction strain^18–23^. Previously, we proposed an SMA spring-based fabric muscle (SFM), and SMA springs comprising 0.5 mm diameter SMA wires were arranged inside the fabrics^21^. The SMA spring-based SFM could lift a mass of 10 kg, approximately 1000 times its own mass of 10 g, with a contraction strain of 50%. In addition, a suit-type wearable robot (STWR) capable of assisting the upper limbs was fabricated by attaching the SFMs to the biceps. After being fitted with the STWR, a mannequin with powerless arms could flex its arms, lift a 4 kg barbell, and keep its arms flexed^17^. However, owing to the SFM’s slow cooling rate, the speed at which the SFM relaxes and the arm extends is slow; thus, it is not easy to repeat the flexion–extension motions of the arm.

SMA spring-based actuators such as SFMs boast numerous advantages such as a high power density and strain; further, they are lightweight and exhibit silent operation. However, their slow cooling rate limits their speed when they actuate repeatedly. Their repeated actuating speed is determined by how rapidly the SMA spring is heated and cooled. The SMA spring’s heating rate can be improved to a level applicable to exosuits by increasing Joule heating power. On the contrary, its slow cooling rate under natural convection cooling (NCC) conditions without an additional cooling method has long been recognized as a limitation of SMA actuators. One method to enhance the cooling rate of SMA involves making the SMA thinner to increase the surface area-to-volume ratio^24,25^. Forced cooling methods such as air cooling^26–28^ and liquid cooling^29–31^, which remove heat while passing an external fluid through the SMA structure, are also possible. Taylor and Au^26^ applied SMA wires to a prosthetic hand as actuators and reduced the relaxation time via forced air cooling (FAC). Park et al.^29^ developed a method of actuating an SMA spring bundle applied to the biceps–triceps of a robotic arm using hot and cold water. They achieved a contraction strain of more than 50% with an actuating frequency of 1-Hz under a 10 kg load. Although liquids can immediately cool SMA springs, they increase the complexity with additional equipment (pump, valve, and liquid tank).

Meanwhile, air cooling structures using fans are simple, lightweight, and economical. Studies have been conducted on which fans should be applied to improve the cooling rate of TCPA or SMA actuators driven by Joule heating. However, the fans could not be directly attached to the actuator because they could interfere with the contraction–relaxation of the actuator. Instead, a separate rigid structure was added to install a fan outside the actuator^26–28^. An additional structure for attaching the fan to the fabric muscle is not suitable for implementation into exosuits because it would inhibit the flexible and light properties of the fabric muscle. Therefore, an integrated fabric muscle capable of active cooling while maintaining the flexibility and lightness of the fabric muscle should be designed to improve the cyclic actuation speed such that it can be used for continuous operation as part of the wearable robotic system.

**Figure 1:**
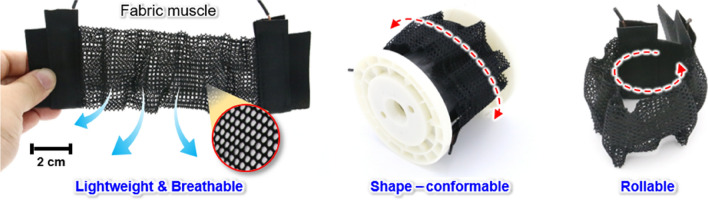
Breathable, light, soft, and flexible fabric muscle.

To this end, a forced fan cooling-integrated fabric muscle (FCFM) is developed that integrates a bundle composed of a large number of SMA springs with high actuation capabilities and improved cooling performance. The proposed FCFM is lightweight, capable of large forces with large contraction displacements, and is capable of sufficient heating and cooling speeds for wearable applications. A bundle composed of a large number of fine-diameter SMA springs with a high surface-area-to-volume ratio is arranged evenly inside a breathable fabric (Fig. 1), and two small fans are attached directly to the fabric. The FCFM making use of a 2.6 g SMA spring bundle achieved stable, repeated contraction–relaxation in ten-second cycles with a displacement of 40 mm for a 4 kg load. An upper limb assistive exosuit using FCFMs was fabricated and worn on a mannequin holding a dumbbell, and the effect of the cooling solution on the speed of the arm’s extension was evaluated. Using fabric muscles for both the biceps and triceps with FAC improves the extension speed by 4.5 times that obtained without fan cooling. Additionally, the assistive force and repeated actuating performance of the exosuits in specific scenarios for repetitive tasks are evaluated, and the applicability of the FCFM as an upper limb assistive exosuit is confirmed.

## Materials and method of fabric muscle

### Fine-diameter SMA springs for fabric muscle

A single SMA spring actuation unit corresponds to a muscle fiber. As shown below, the SMA spring stiffness (F) is determined based on the number of coil turns (n), the wire diameter (d) to spring diameter (D) ratio, and the displacement of the SMA spring (δ), and the shear modulus (G).

1 $$ {\text{F}} = \left( {\frac{{Gd^{4} }}{{8D^{2} n}}} \right)\delta $$

**Figure 2:**
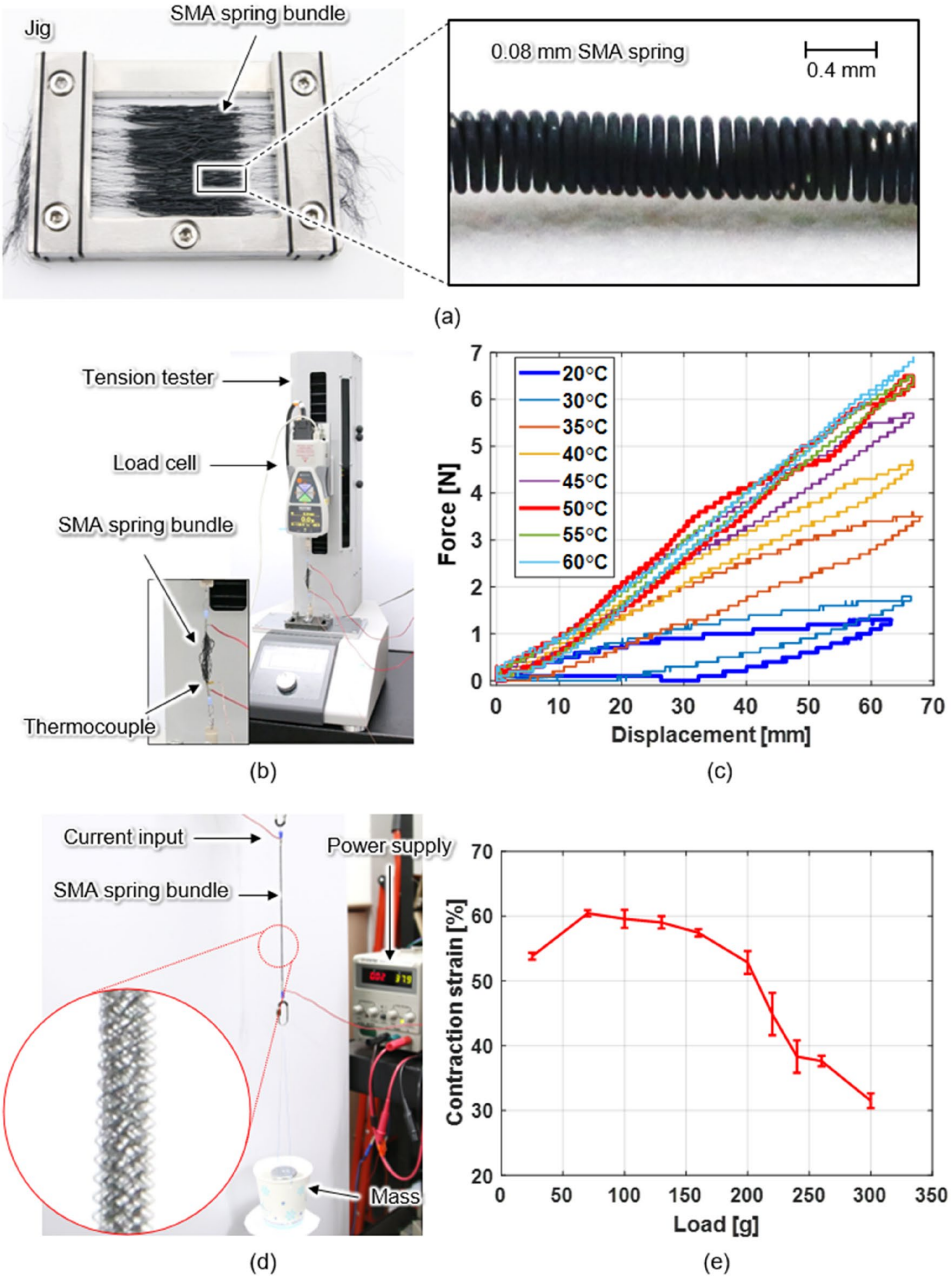
Fabrication and performance evaluation of the SMA spring bundle. (**a**) Aligned SMA springs. (**b**) Experimental setup for isothermal condition tests. (**c**) Isothermal force–displacement curve of SMA spring bundle with respect to heating temperature. (**d**) Experimental setup for measuring the load capacity of SMA spring bundle. (**e**) Relationship between load and contraction strain of the SMA spring bundle.

The parameters of the SMA spring fabricated in this study are presented summarized in Table 1. The fabric muscle force increases in proportion to the number of embedded SMA springs^21,32^. A NiTi SMA wire with an austenite finish transition temperature of 48 °C was used to fabricate SMA spring bundles (Fig. 2a). This study utilized a 0.08 mm- diameter SMA wire, improving the surface area-to-volume ratio 6.25 times over the 0.5 mm- diameter SMA spring^21^. The fabrication process for the 0.5 mm- diameter SMA spring is described in a previous study^21^. A fabric muscle employing 0.08 mm-diameter SMA springs, however, would require a far higher number of SMA springs to have the same load capacity as a fabric muscle using 0.5 mm-diameter SMA springs^21^. Accordingly, a new process capable of fabricating a large amount of SMA springs was used. The procedure to fabricate the SMA springs is as follows. A NiTi SMA wire is wound in a coil-spring shape on a core wire with a diameter of 360 μm using a coiling machine. The core wire continuously wound with the coil springs is wound on the bobbin. Then, for the memorization of the coil-spring shape, the SMA spring wound on the bobbin is annealed in an electric furnace at 400 °C for 30 min. Subsequently, the annealed SMA springs are cut into coil units and aligned on the jig (Fig. 2a). Finally, to remove the core wires in the SMA springs, immerse the jig in the hydrochloric acid solution for 30 min to melt.

**Table 1:** Specification of SMA spring.

Item	Unit	Value
SMA wire diameter, *d*	mm	0.08
SMA spring coil diameter, *D*	mm	0.44
Number of spring coil turn, *n*	turn	325
SMA spring coil length	mm	26
SMA spring leg length	mm	60
Weight (spring coil + leg)	g	0.013

The stiffness of the SMA spring under isothermal conditions was measured as shown in Fig. 2b. Twenty-five SMA springs were bundled and fixed to a tension tester. A thermocouple was attached to the SMA spring bundle, and the input current was adjusted and controlled by temperature feedback (Fig. 2b). The displacement and force generated by the SMA spring bundle as it was pulled were measured from 20 to 60 °C (Fig. 2c). The slope of the displacement–force curve gives the stiffness of the SMA spring bundle at a specific temperature^21^. A low-temperature stiffness of 0.82 N/m and high-temperature stiffness of 3.89 N/m of one strand of the SMA spring was measured. Next, the contraction strain of the SMA spring bundle was measured depending on the load (Fig. 2d). Ten SMA springs were bundled; one end was fixed to the frame, and a load mass was hung on the other end, after which power was supplied to contract the SMA spring bundle. Here, 9.3 W of power was supplied for 1.5 s. The mass was varied between 25 and 300 g, and the contraction strain of the SMA spring bundle was measured five times under each mass condition (Fig. 2e). The contraction strain was calculated as follows:

2 $$ {\text{Contraction strain}} \left[ \% \right] = \frac{dl}{{l_{0} }} \times 100 $$

where $l_{0}$ is the initial length of the SMA spring bundle extended by the mass, and dl is the contraction displacement of the SMA spring bundle by heating.

Under a 70 g load, a maximum contraction strain of 60% was generated, and the contraction strain remained above 50% up to a load of 200 g; however, it rapidly decreased to less than 50% under loads of 220 g or more. The load capacity of 10 bundles of SMA springs to cause a contraction strain of more than 50% was 2 N.

### Design and fabrication of fabric muscle

To determine the specifications of the fabric muscle for an upper limb assistive exosuit, the force–displacement required for the flexion–extension motion of a 1-degree-of-freedom arm was evaluated (Fig. 6b). We assume that (1) the arm is holding a load such as a dumbbell; (2) two fabric muscles are attached to the biceps and triceps in the upper arm in an antagonistic structure that can contract and relax, and the forearm is flexed and extended. Tahe force requirements of the fabric muscles attached to the biceps and triceps were calculated as 80 N and 40 N, respectively.

**Figure 3:**
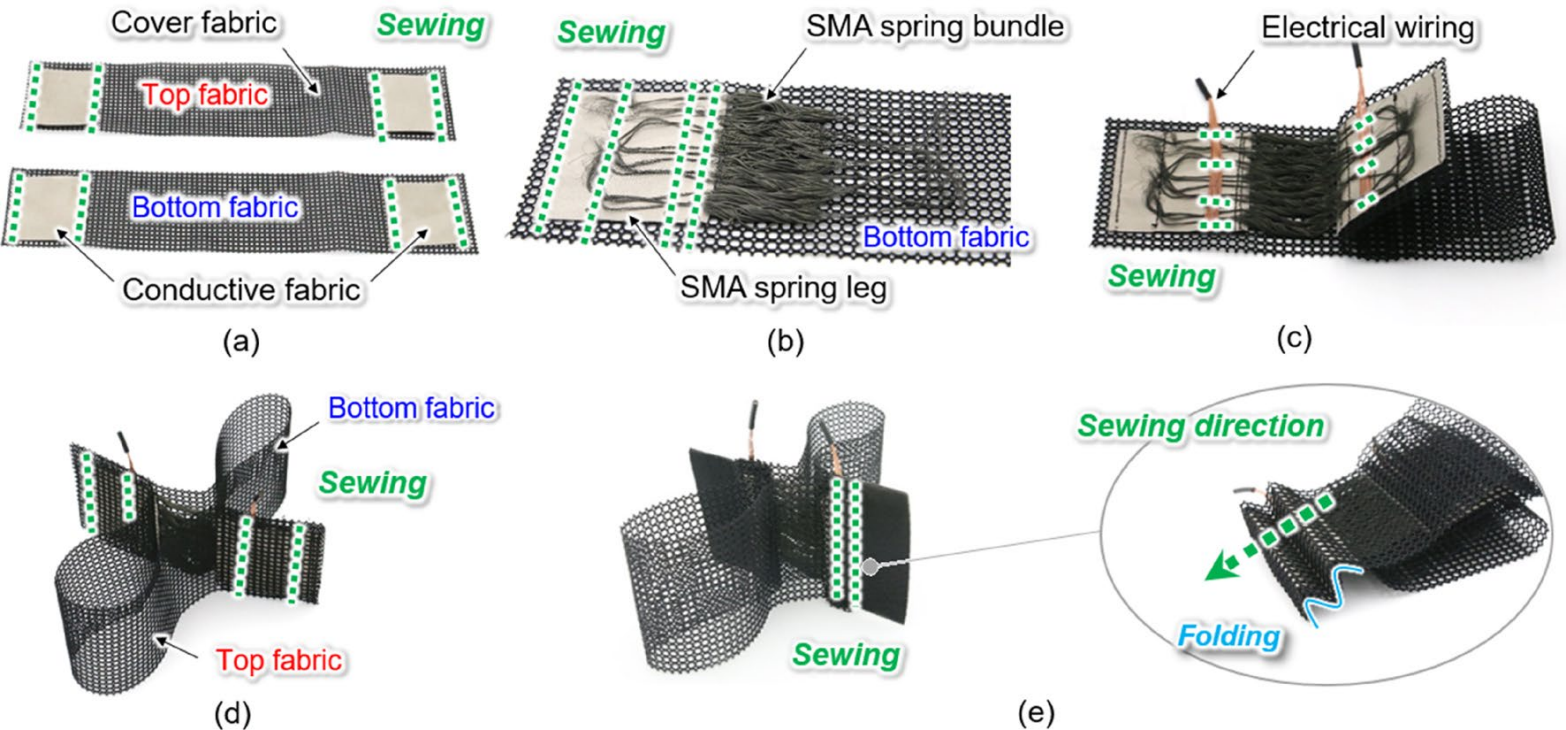
Fabric muscle fabrication process. (**a**) To electrically connect the SMA springs, the conductive fabric (50 mm $\times$ 50 mm) is sewn (green dotted lines) and fixed to the cover fabric (both top and bottom fabrics, width 60 mm, height 300 mm). (**b**) 200 SMA springs are aligned on the cover fabric (bottom fabric). SMA spring legs are arranged above and below the conductive fabric and sewn fixed so that electricity can flow to the SMA springs. (**c**) Electrical wires are arranged above and below the conductive fabric and sewn fixed to supply external power to the SMA springs. (**d**) Cover fabrics (both top and bottom) are overlapped so that the conductive fabrics align and then sewn. (**e**) Conductive fabric part with the SMA spring legs fixed is folded twice and fixed with sewing.

The fabric muscle is comprised of SMA spring bundles, conductive fabrics, cover fabrics, and electrical wiring (Fig. 3). In tandem with the conductive fabric, the wiring supplies current from the external power source to the SMA springs. Thin conductive fabrics (1168, Adafruit, USA) were folded together with both ends of the SMA springs and sewn, enabling the conductive fabric to fix the SMA spring bundles and function as a soft electrode. The SMA spring legs were arranged on the conductive fabric sewn on the cover fabric, and then the fabrics and SMA spring legs were folded and sewn together, allowing for the integration of a large number of SMA springs with the conductive fabric. The cover fabric protects the SMA spring bundle, electrically insulates it, and limits the maximum extension length of the fabric muscle. A piece of mesh fabric with excellent ventilation was selected as a cover fabric material.

The total resistance of the fabric muscle is determined by the combination of serial and parallel connections between the SMA springs. To produce the same Joule heating power, if the number of parallel connections between SMA springs is large, the total resistance decreases; thus, the supply current must be high. If the number of series connections is large, the total resistance increases; thus, the supply voltage must be high. Therefore, for wearable robots using a portable battery with limited capacity, the total resistance of the fabric muscle must be determined considering the battery specifications for the power supply^21^.

The fabric muscle for upper limb assistance, which is suitable for the biceps and triceps, has a width of 60 mm and a length of 200 mm (Fig. 1). The actual actuation part is 180 mm, which corresponds to the coil part of the SMA spring and is the extended length obtained after applying the load. If more force is required, multiple overlapping fabric muscles can be used to easily increase the total assistive force. The mass of the SMA springs alone was 2.6 g, and the total mass including the cover fabrics, electrical wiring, and conductive fabrics is 18 g. The fabric muscle is soft, flexible, light, and can be rolled up like fabric or folded like paper.

### Performance evaluation of fabric muscle

#### Natural convection cooling

**Figure 4:**
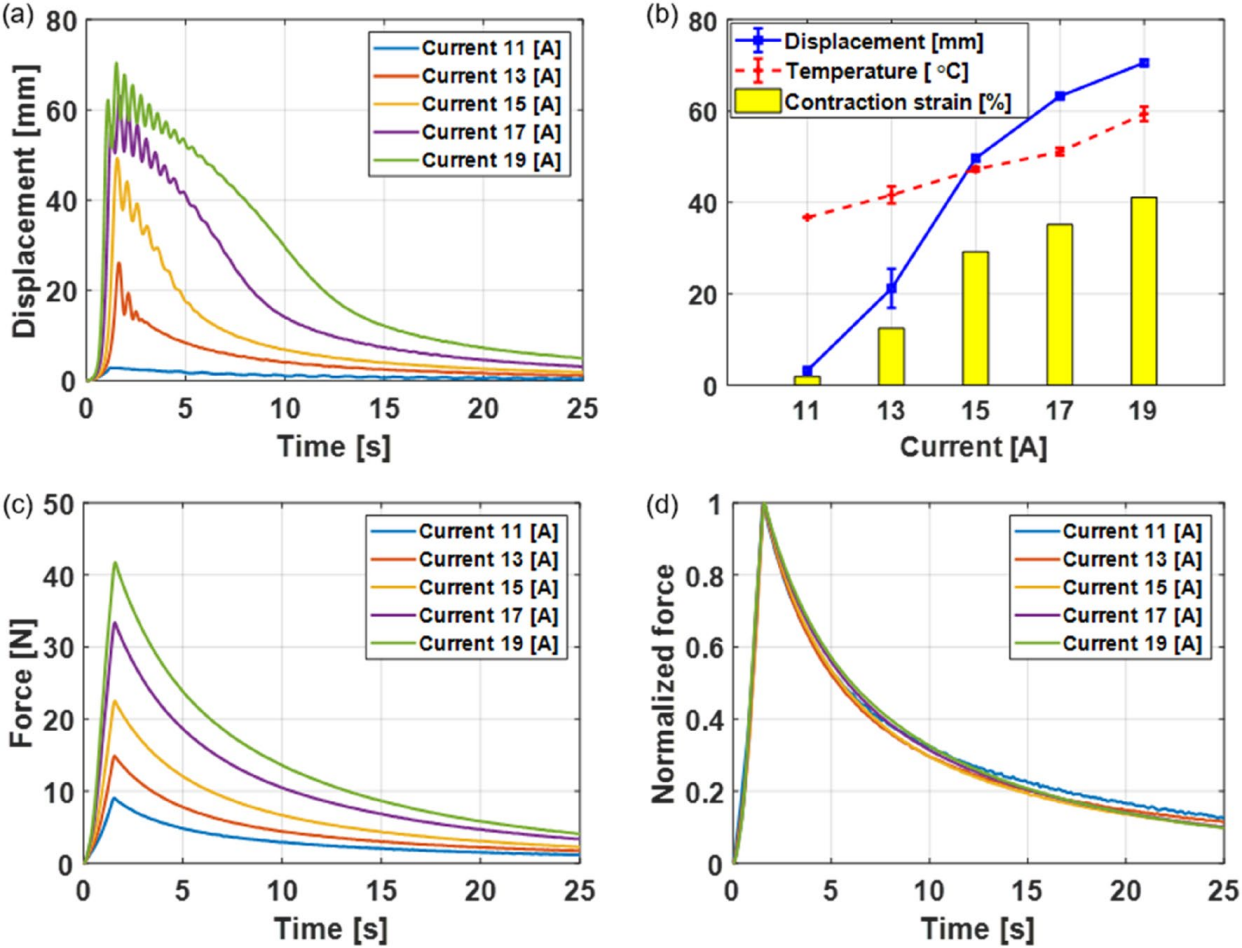
Performance evaluation of fabric muscle under NCC. (**a**) Changes in displacement of fabric muscle measured during Joule heating and NCC according to input current under 4 kg load condition. (**b**) Comparison of maximum displacement (blue line), contraction strain (yellow bar-graph), and heating temperature (red dot-line) of fabric muscle according to input current. (**c**) Generated force of the fabric muscle depending on the input current under the isometric condition. (**d**) Normalization result of the force data measured in (**c**).

The significant performance indicators of fabric muscles are the generating force, displacement, contraction strain, and actuating speed. These were evaluated under NCC conditions. First, the maximum displacement and maximum temperature of the fabric muscle depending on the supply current were measured. The upper end of the fabric muscle was fixed to a frame, and a 4 kg mass was hung at the lower end. A laser displacement sensor (LK-G500, Keyence Corp., Japan) was installed above the fabric muscle, and a thermocouple (TT-K-30-SLE, Omega Engineering Inc., Republic of Korea) was attached inside the fabric muscle. Current from 11 to 19 A was supplied by a 14.8 V portable battery (Anhui Enrichpower Battery Co., LTD, China). The length, width, height, and weight of the battery are 70 mm, 34 mm, 37 mm, and 175 g, respectively. The current was applied for 1.5 s, and the relaxation time of the fabric muscle was measured under NCC. The fabric muscle relaxed to 80% and 90% of the initial length after 14.5 s and 18.8 s from the start of NCC, respectively (Fig. 4a). Figure 4b compares the fabric muscle displacement, contraction strain, and maximum heating temperature depending on the supply current. As the supply current increased, both the displacement and maximum heating temperatures of the fabric muscle increased. When the supply current was 19 A, the displacement was 70.4 mm, the contraction strain was 39.1%, and the maximum temperature increased to approximately 60 °C. Isometric tests were conducted to measure the force generated by the fabric muscle. Both ends were fixed after the fabric muscle was extended to a length of 180 mm. Five current values ranging from 11 to 19 A were supplied for 1.5 s each (Fig. 4c). The highest magnitude of the produced force grew in proportion to the supply current, reaching 41.7 N at 19 A.

3 $$ F_{norm} = \frac{F}{{F_{max} }} $$

Figure 4d shows the normalized force ($F_{norm}$) of the generated force ($F$) according to each supply current using (3). Here, the maximum force ($F_{max}$) is generated 1.5 s after heating under each current condition; the slopes of increasing forces due to heating were similar despite different supply current conditions, and the slopes of decreasing forces due to NCC were also similar. The relaxation time is defined as the time elapsed until the force decreases to a specific ratio of the maximum force. The force decreased steeply up to 60% of the maximum force with a relaxation time of 5.9 s. After this point, the fabric muscle force decreased slowly; the 80% relaxation time was 13.6 s, and the 90% relaxation time was 23.4 s.

In the case of relaxation via NCC, the 80% cooling rate was achieved after 14.5 s, which is considerably slower than the time observed for the skeletal muscle. Applying this fabric muscle to an exosuit can provide assistive force for flexion motions of the arm to lift heavy objects or to maintain the arm in a flexed position while holding an object. However, for extension motions, the fabric muscle temperature gradually decreases. Therefore, the residual contraction force of the fabric muscle can interfere with arm extension. The relaxation speed of the fabric muscle must be improved such that the exosuit can be useful in applications with frequently repeated flexion–extension motions.

#### Forced-air cooling fan-integrated fabric muscle

**Figure 5:**
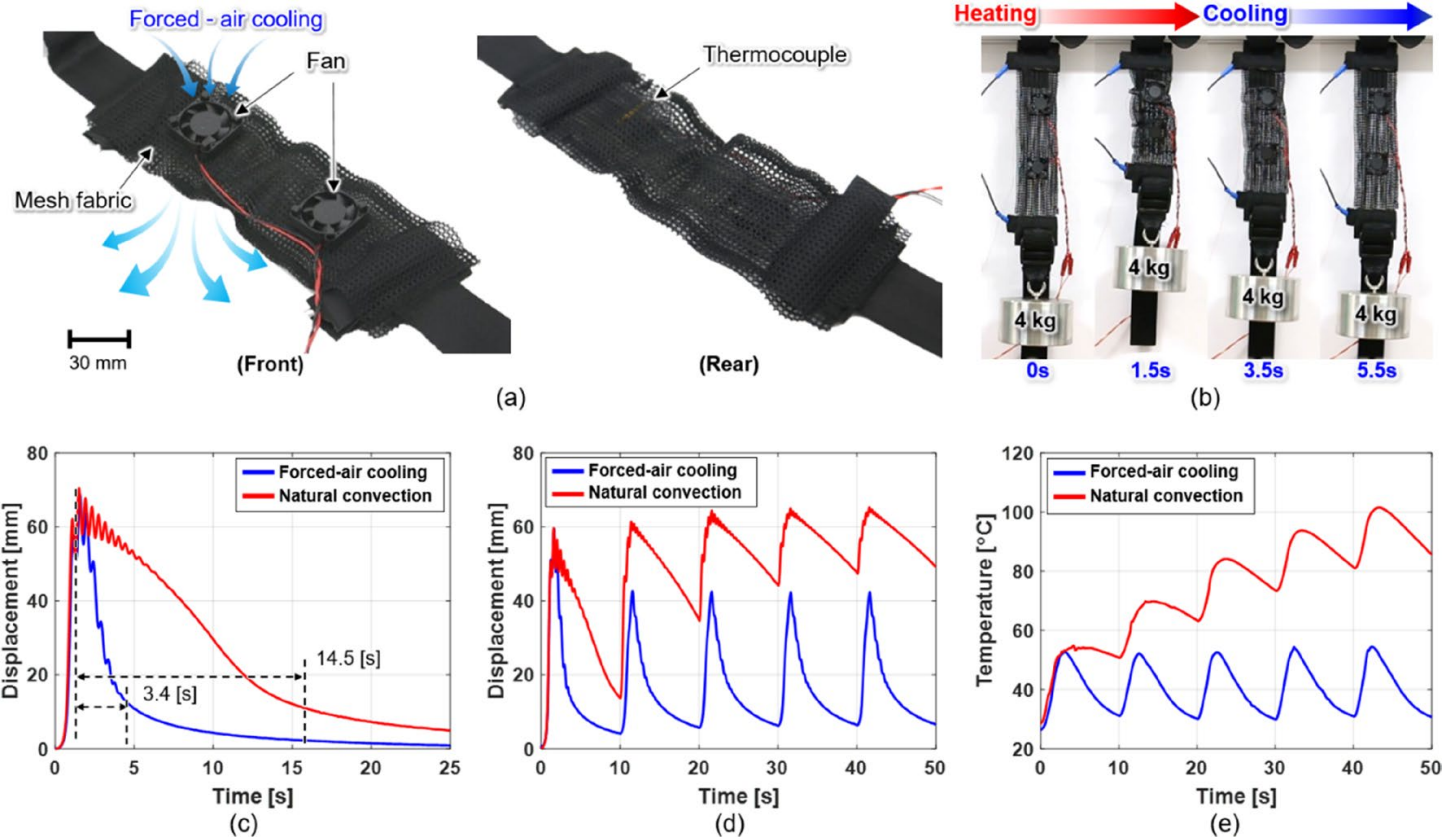
Performance evaluation of fabric muscle under FAC. (**a**) Front view of fabric muscle attached with two small fans for forced air cooling (left). A thermocouple is attached inside the fabric muscle to measure the actuation temperature of the fabric muscle (right). (**b**) The upper end of the fabric muscle was fixed to a frame, and a 4 kg mass was hung at the lower end. Sequence of contraction and relaxation by 19 A and 1.5 s Joule heating and FAC. (**c**) Comparison of relaxation rates of fabric muscle measured during Joule heating and cooling under different cooling conditions (see Video S1). Comparison of change in (**d**) displacement and (**e**) temperature when performing contraction-relaxation at actuating frequency of 10 s under NCC and FAC.

Two small fans—with specifications summarized in Table 2—are attached to one cover of the fabric muscle (Fig. 5a) to apply FAC to the SMA springs. Since the upper and lower fabric muscle covers comprise a mesh with good ventilation, the air supplied by the fans passes through the front cover, cools the SMA springs inside, and then exits through the rear cover. The fans turn on only to supply cooling air to relax the fabric muscle in the contracted state after heating (Fig. 5b). The fans were switched on and off by MOSFET-based (IRF-840, Vishay Intertechnology Inc., USA) control. The fan's wind speed input to the fabric muscle was 4.5 m/s, and the wind speed exiting the rear side after passing through the fabric muscle was reduced to 1.3 m/s. The area ratio of the fan to the fabric muscle was 15% in a relaxed state, increasing to 25% when the fabric muscle was 39.1% contracted. In a contracted state, the space between the coils of the SMA springs was narrow and dense, enabling sufficient cooling by the fan-generated airflow. As the two attached fans were small, they did not interfere with the contraction of the fabric muscle. The cooling performance of the FCFM under NCC and FAC was compared based on the following: (1) relaxation time after one contraction. (2) Change in displacement and temperature during repeated actuation.

**Table 2:** Specification of cooling fan.

Item	Unit	Value
Size (width $\times$ height $\times$ thickness)	mm	30 $\times$ 30 $\times$ 6
Weight	g	6
Rated voltage	V	5
Rated current	A	0.05
Input power	W	0.25
Speed	rpm	6000
Maximum air flow	${\text{m}}^{3}$/min	0.057
Maximum noise	dB	13.26

A 19 A Joule heating current was supplied for 1.5 s, and the fabric muscle contracted by 70.4 mm. The relaxation step began immediately after the fabric muscle contracted, and the fan was activated to cool the fabric muscle (Fig. 5c). Table 3 compares the relaxation times of the fabric muscles under natural and FAC. The 60% relaxation time decreased by 82.7% to 1.5 s under FAC. Compared with NCC alone, the FAC better improved the relaxation rate of the fabric muscle. The contraction–extension displacement and temperature of the fabric muscle were measured and compared (Fig. 5d, e). A 17 A Joule heating current was supplied for 1.5 s and then cooled for 8.5 s to repeatedly actuate the fabric muscle in ten-second cycles. Under NCC, a contraction displacement of 60 mm occurred in the first actuation; however, the contraction displacement was reduced to 46.7 mm in the second actuation because the fabric muscle was not sufficiently relaxed during the cooling time of 8.5 s. Because of the insufficient cooling time, as the number of cycles increased, the relaxation displacement decreased, and accordingly, the contraction displacement gradually decreased. In this process, although the fabric muscle temperature decreased in the cooling section, the minimum and maximum temperatures in one cycle continued to rise owing to insufficient cooling and repeated Joule heating. The maximum temperature increased up to 100 °C in the fifth Joule heating cycle. In contrast, under FAC, stable repeated actuation with a constant contraction displacement of 40 mm corresponding to a contraction strain of 22.2% was possible. Under FAC, 8.5 s corresponds to 93% relaxation time; as such, the fabric muscle can be sufficiently relaxed, and even if Joule heating is applied repeatedly, a constant contraction displacement can be achieved. Here, the temperature of the fabric muscle was maintained in the range of 35–55 °C. When adding two fans weighing a combined 12 g to the 18 g fabric muscle, the mass of one FCFM increases to 30 g; however, it is still light considering the improved cooling rate. When the exosuit supports tasks performed in short ten-second cycles under NCC, it may be dangerous to the skin due to the continuously rising temperature. Therefore, to shorten the period of task cycles that the exosuit can assist, a fabric muscle with FAC should be used.

**Table 3:** Comparison of cooling time for NCC and FAC.

Recovery ratio [%]	Cooling time [s]		Cooling time reduction* [%]
	NCC, $t_{N}$	FAC, $t_{F}$	
60	8.7	1.5	82.7
80	14.5	3.4	76.5
90	18.8	5.6	70.2

*Cooling time reduction = $100 - \left\{ {\left( {100 \times t_{f} } \right){/}t_{n} } \right\}.$

### Evaluation of repeated actuation of upper-arm assistive exosuit

**Figure 6:**
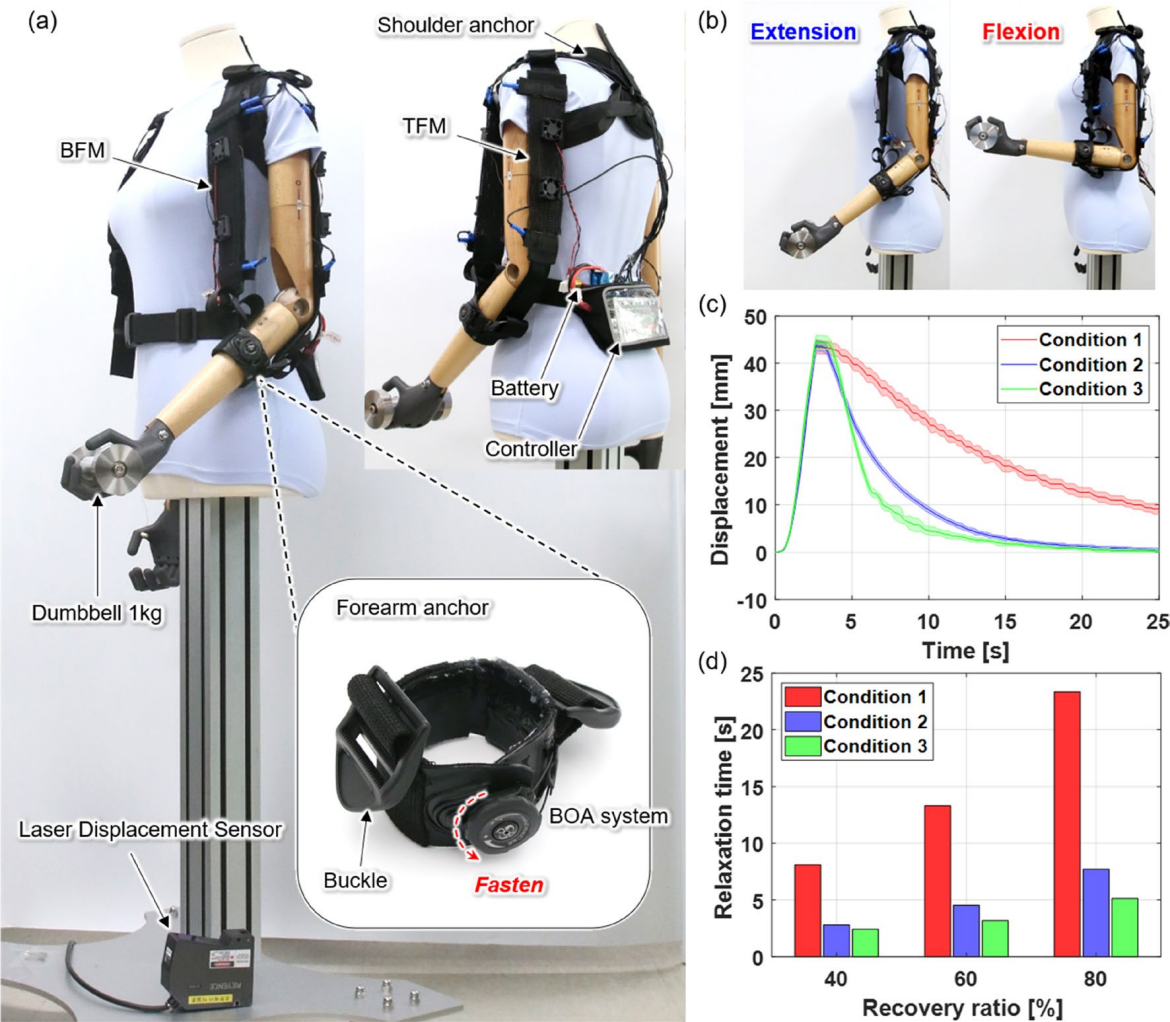
Assistance of the mannequin’s flexion–extension motions by the exosuit. (**a**) Experimental setup. The shoulder anchor was made with length-adjustable straps. The forearm anchor uses a BOA (BOA Technology Inc., Denver, USA) fastener to enable easy loosening and tightening. The upper ends of the BFM and TFM are connected to the shoulder anchor, and the lower ends are connected to the forearm anchor. A laser displacement sensor installed on the floor measured the contraction-relaxation displacement of the fabric muscle. A thermocouple inside the BFM and TFM to measure the temperature. (**b**) The mannequin arm lifts a 1-kg dumbbell with the assistance of the exosuit. When the BFM relaxes and the TFM contracts, the mannequin arm is in an extended state (left), and when the BFM contracts and the TFM relaxes, the arm becomes is in a flexed state (right). (**c**) Comparison of flexion and extension responses under three different conditions (see Video S2). (**d**) Relaxation times for the three different recovery ratios of BFM.

The upper limb assistive exosuit consists of a shoulder anchor, forearm anchor, biceps fabric muscle (BFM), triceps fabric muscle (TFM), battery, and controller (Fig. 6a). If solely the BFM is attached to the exosuit without the TFM, the flexion and extension motions of the mannequin arm can be realized by contracting the BFM through the current supply and relaxing the BFM through NCC or FAC. However, similar to the antagonistic structure of biceps–triceps in the human arm, the extension speed of the arm can be increased by attaching the TFM to the exosuit and contracting it. The BFM comprises two overlapping 40 N-fabric muscles and can exert a maximum force of 80 N, whereas the TFM uses only one 40 N-fabric muscle. The battery and control board pack can be attached to the lower back. In total, the exosuit weighs 0.57 kg.

By mounting the exosuit on a mannequin that does not have its own force to lift or lower a load, the exosuit’s assistive force effect can be evaluated. A 1 kg dumbbell was attached to the hand of the exosuit-wearing mannequin. The BFM and TFM of the exosuit were arranged antagonistically. Therefore, the more the stiffness was reduced by lowering the temperature of the fabric muscle opposite the actuating fabric muscle, the greater force was exerted on the arm by the actTuating fabric muscle while minimizing loss. The flexion–extension motion assistance performance of the exosuit (Fig. 6b) was evaluated under the following three conditions: (1) biceps-only + NCC. (2) Biceps-only + fan cooling. (3) Biceps and triceps + fan cooling.

4 $$ D_{norm} = \frac{D}{{D_{max} }} $$

First, the contraction–relaxation displacement of the BFM over time as the mannequin arm performed one flexion–extension motion was measured (Fig. 6c). The three conditions produced identical contraction displacements of 47 mm and contraction strains of 26.1%; however, the difference in the relaxation rate was evident. The displacements ($D$) under the three conditions were normalized ($D_{norm}$) using (4), and then the relaxation times of the BFM were calculated (Fig. 6d). The maximum displacements ($D_{max}$) were generated at 3 s after heating under all three conditions.

Under Condition 1, the time elapsed for the BFM to recover 40% of the maximum contraction displacement was 8.1 s, and the 80% relaxation time was 23.3 s. Under Condition 2, the 40% and 80% relaxation times were 2.7 and 7.7 s, respectively; thus, the extension speed nearly tripled under Condition 1. Under Condition 3, in which the TFM also contracts in the extension motion, the 80% relaxation time was 5 s; hence, the extension speed improved by approximately 4.5 and 1.5 times compared to Conditions 1 and 2, respectively. These results demonstrate that the arm’s extension speed can be significantly improved by adding fan cooling to the fabric muscle and can be further enhanced if the contraction of the TFM assists in arm extension.

**Figure 7:**
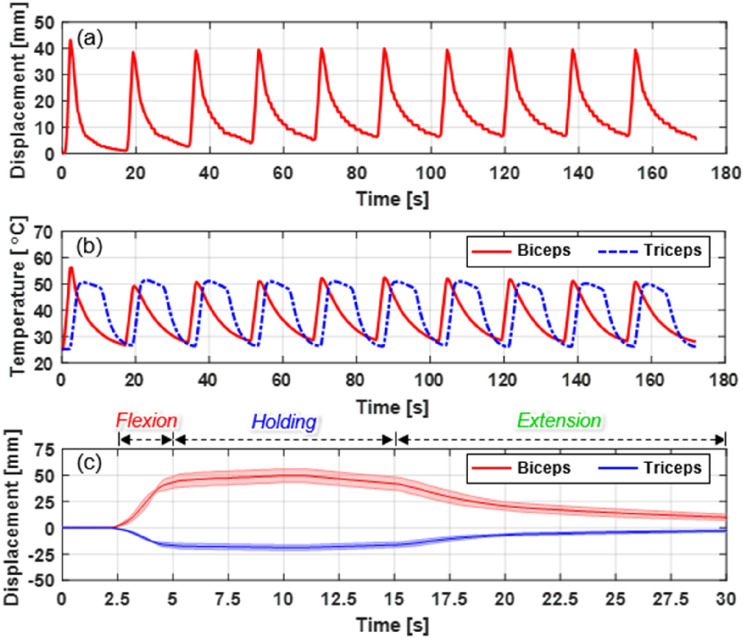
(**a**) BFM displacement when repeating flexion–extension motion 10 times in Condition 3. (**b**) Temperature change of BFM and TFM when repeating flexion–extension motion in Condition 3. (**c**) Displacement of BFM and TFM during temperature control.

The arm flexion–extension motions were repeated, and the contraction–extension displacement and temperature of the BFM and TFM were measured and compared. Under Condition 3, in which the arm’s flexion–extension speed was the fastest in the previous experiment, the flexion–extension was repeated ten times in eighteen-second cycles. A current of 10 A was supplied only to the BFM for 3 s to flex the mannequin arm, and then the arm was immediately extended by simultaneously fan cooling the BFM and supplying 10 A of current to the TFM for 3 s. Subsequently, the heated TFM was fan-cooled for 12 s. As the number of cycles increased, the initial starting position of the BFM was offset by approximately 5 mm; however, stable displacement was observed in the range of 0–40 mm for ten cycles (Fig. 7a, see Video S3). Furthermore, both the BFM and TFM were heated/cooled within a limited temperature range of 25–50 °C (Fig. 7b). It was possible to repeat the arm flexion–extension motions more than 120 times with a portable battery.

As the contraction displacement of the fabric muscle is generated in proportion to the temperature of the SMA springs, it can be maintained constant by controlling the heating temperature. If the BFM can maintain the contraction, it can support the flexion of the arm and assist the wearer in maintaining the load on the arm. A thermocouple attached to the BFM fed back its temperature to adjust the amount of current by the proportional-integral control. An experiment was conducted in which the mannequin maintained a flexion state holding a 1-kg dumbbell (Fig. 7c). When the BFM temperature was maintained at 60 °C after BFM contraction, the arm flexion state was maintained with a displacement of 50 mm. Then, the BFM was cooled by natural convection while simultaneously supplying a current of 11 A to the TFM for 3 s to perform extension. Finally, all exosuit power was turned off, and both BFM and TFM were cooled by natural convection (see Video S4). Therefore, the exosuit could assist both in lifting the load and in holding the lifting state. A continuous current must be supplied to maintain the lifting state. The mechanism from the previous study^33^ could be utilized to maintain the lifting state without supplying current and extend the operating time of the battery.

### Evaluation for repetitive task scenarios

**Figure 8:**
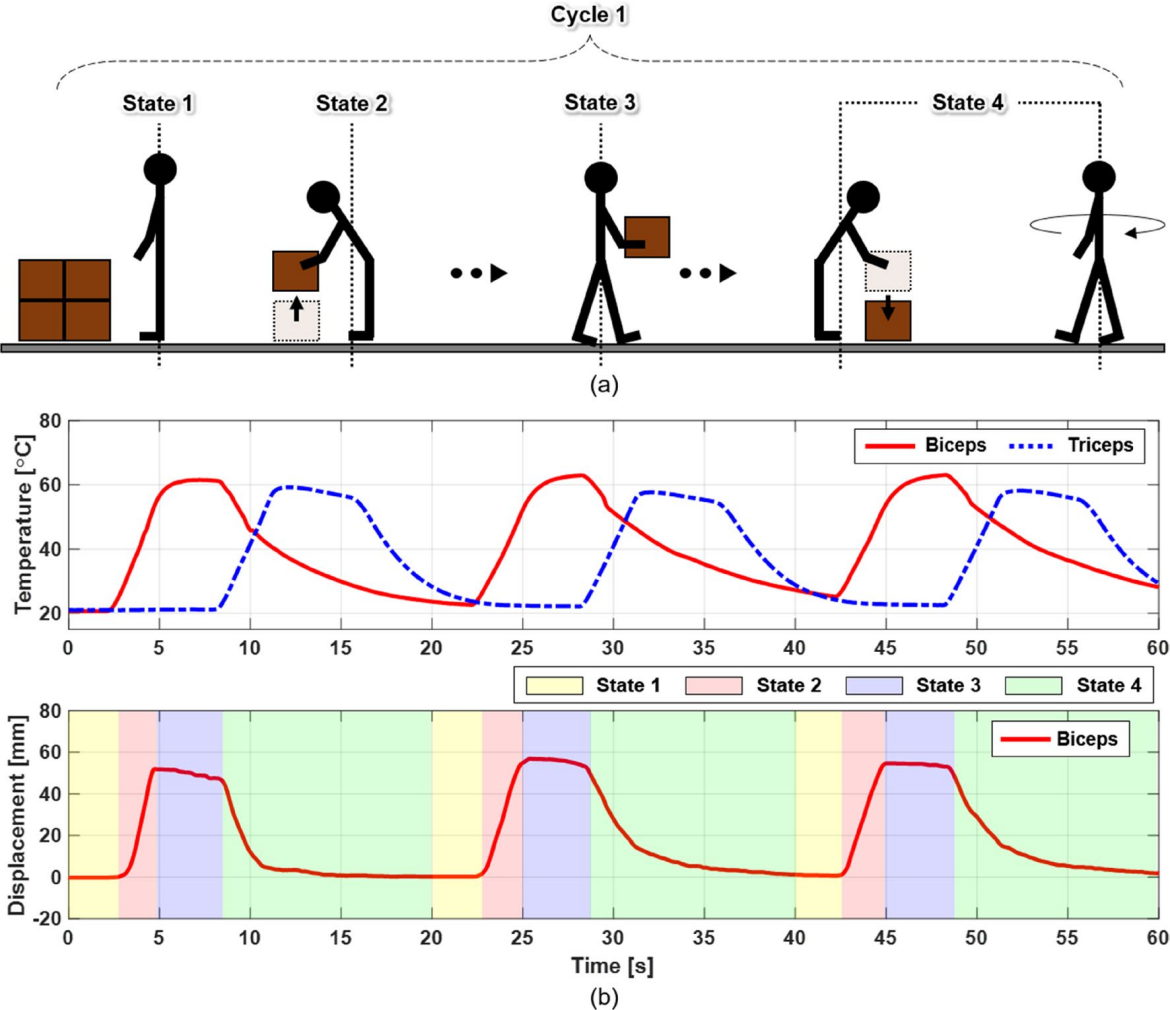
Example of strength-assisted task performed by person wearing exosuit applying fabric muscle (see Video S5). (**a**) The worker prepares to lift the object with both the BFM and TMF of the exosuit relaxed in a loading area (State 1: 2 s). As the worker flexes his arm and lifts the object, the BFM of the exosuit contracts to support the muscle strength (State 2: 3 s). The BFM is then controlled to a constant temperature, and the worker moves to an unloading area while maintaining the flexion state of the arm holding the object (State 3: 3 s). Finally, the worker extends his arms, lowers the object into the unloading area, and returns to the initial position (loading area) to carry the next object (State 4: 12 s). (**b**) Measured temperature (top graph) and displacement (bottom graph) of BFM and TFM according to the state.

This section examines specific task scenarios involving repetitive motions. As shown in Fig. 8a, a worker, simulated by a mannequin wearing an exosuit lifts and carries an object in a repeated cycle of 20 s, in which the motions of the worker are divided into four states. The exosuit was operated for a total of three cycles, and the results are shown in Fig. 8b. To perform the lifting motion in State 2 after the preparation stage of State 1, a current of 10 A was applied to the BFM for 3 s, and the BFM was heated from 25 °C to 60 °C. Here, a contraction displacement of 50 mm occurred in the BFM, and the mannequin arm flexed and lifted the dumbbell. Then, as shown in Fig. 8b, the temperature was controlled at 60 °C for 3 s to maintain the flexion state of the mannequin arm holding the dumbbell (State 3). Finally, the BFM was forcibly cooled and a current of 10 A was applied to the TFM for 3 s to extend the arm (State 4). When the temperature of the heated BFM was reduced by FAC, the TFM contracted while being heated to a maximum of 58.7 °C, and the BFM was rapidly relaxed to its initial length. Accordingly, the mannequin arm reached its initial extended position, immediately after which the heated TFM was forcibly cooled to prepare for the next repeated motion. This experiment demonstrated that the FCFM applied to the exosuit can yield good heating–cooling performance for repetitive tasks in twenty-second cycles (see Video S5).

**Figure 9:**
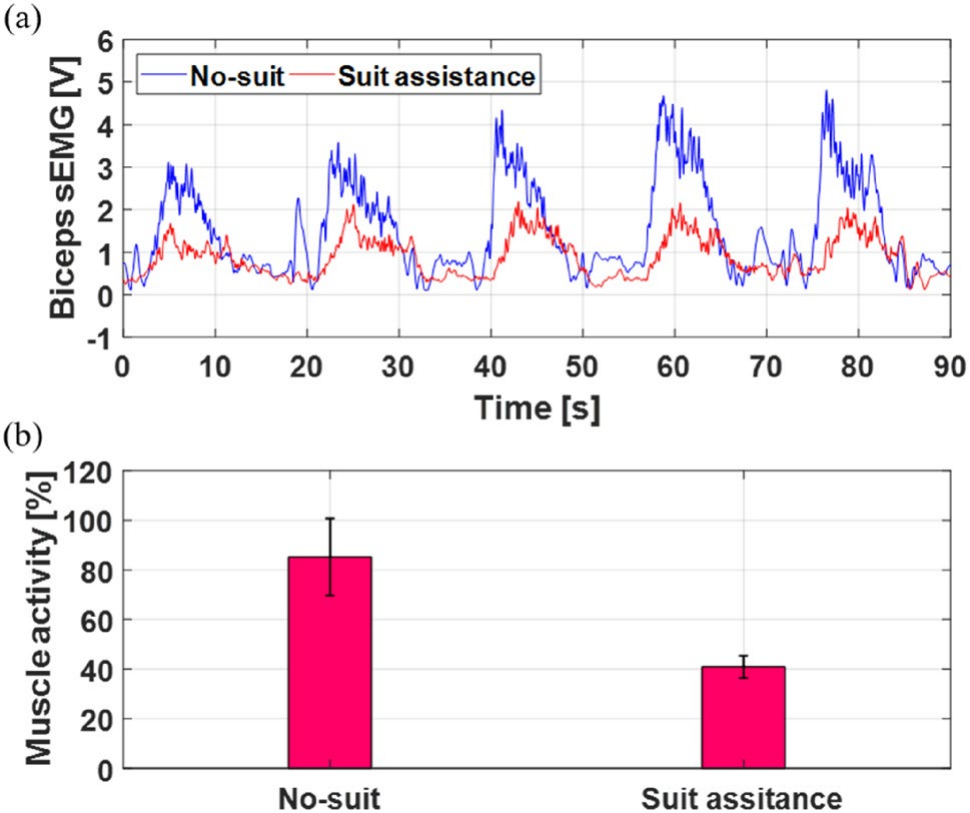
Evaluation of the assistive performance for exosuit. (**a**) Comparison of sEMG signals obtained from the biceps of a representative subject between No-suit and Suit assistance conditions. (**b**) Comparison of muscle activity of subjects between No-suit and Suit assistance conditions.

One of the authors evaluated the assistive performance of the proposed fabric muscle by wearing an exosuit making use of the fabric muscle and repeatedly lifting a dumbbell (The experimental protocol was approved by the Institutional Review Board at Chungnam National University (202101-SB-013-01). We confirmed that all experiments were performed in accordance with relevant guidelines and regulations.). The author wearing the exosuit measured the sEMG (surface electromyography) signals in the biceps brachii by using an sEMG sensor (MyoWare Muscle Sensor AT-04-001, Advancer Technologies, NC, USA) while lifting and lowering a 3 kg dumbbell 5 times in a cycle, similar to the scenario in Fig. 8a. The sEMG signals of the biceps were compared with and without the assistance of the exosuit (Fig. 9a). Without assistance will be referred to as the “No-suit” condition, and in this condition the wearer's biceps required additional muscle activation resulting in fatigue as the peak of the biceps sEMG signal gradually increased and reached a maximum of 4.8 V. However, in the Suit assistance condition, the peak value of the sEMG was maintained below 2 V even through repeated motions. With the help of the exosuit, the wearer's muscle activity decreased by 51.9%, making it possible to perform repetitive motions for an extended period of time (Fig. 9b). In the future, we plan to evaluate the assistive performance of the exosuit on a large number of subjects with in various scenarios.

### Statement of consent

We consent for publication of identifying information/images in an online open-access publication.

## Conclusion

This study focused on the design, fabrication, and performance evaluation of a fabric muscle, that can produce a stable cyclic actuation within a specific temperature range while increasing the repeated actuating speed. Although increasing the speed of SMA springs through the application of higher electric power through Joule heating is straightforward, the integration of FAC with an SMA spring bundle for portable applications has not yet been demonstrated and is necessary to reduce the cooling time sufficiently for continuous operation in wearable applications. Few examples of SMA spring bundles have been presented due to implementation issues and cooling speed issues that compound as the number of springs is increased. This research presented a self-contained lightweight (30 g) FAC fabric muscle that contains up to 200 SMA springs to produce a large force (> 40 N) with a large contraction displacement (> 60 mm) and uses FAC to significantly increase the cooling speed of the muscle. This results in a fabric muscle which is highly suitable as a wearable robotic device. Indeed, the force, contraction displacement, heating speed, and cooling speed are sufficient to assist a worker using the FCFM as part of a wearable exosuit for strength-assisted tasks without waiting for cooling between operations. The muscle activity of a subject with the assistance of the exosuit was reduced by about 51.9% compared to the condition without the assistance of the exosuit. This result indicates that the support of the exosuit reduces muscle fatigue and assists the wearer while doing physical activities for a longer period of time.

SMA springs made from SMA wire with a diameter of 0.08 mm, which is thinner than a hair, were combined with a mesh fabric cover with good ventilation. This configuration promoted the release of heat generated from the SMA spring bundle. Moreover, by attaching small fans directly to the fabric cover of the fabric muscle, the cooling effect was maximized while maintaining the flexibility and wearability of the fabric muscle. This FAC reduced a 90% relaxation time to 5.6 s, 70.2% compared to 18.8 s for the NCC. In addition, the use of a smaller-diameter SMA wire increases the surface area-to-volume ratio of the SMA spring bundle, and the cooling rate can be further improved.

An upper limb assistive exosuit applying the FCFM was demonstrated. This exosuit was attached to a mannequin, and the antagonistic actuation of the FCFMs of the biceps and triceps effectively increased the cyclic actuating speed of the mannequin arm. In this study, to improve the extension speed of mannequins with no muscle strength, an exosuit was fabricated with an additional FCFM attached to the triceps. However, for an actual human worker wearing the exosuit, the TFM may be unnecessary because the strength of his own triceps may be sufficient to improve the extension speed.

These fabric muscle-based exosuits can be utilized in various applications, including rehabilitation, industry, military, and leisure. In the future, we will investigate the assistive effect produced by supporting actual workers' task motions to further enhance the exosuits.

## Supplementary Information

Supplementary Information 1.

Supplementary Information 2.

Supplementary Information 3.

Supplementary Information 4.

Supplementary Information 5.

Supplementary Information 6.

Supplementary Information 7.

Supplementary Video 1.

Supplementary Video 2.

Supplementary Video 3.

Supplementary Video 4.

Supplementary Video 5.

Supplementary Legends.

## Data Availability

All data in the paper are included in the Supplementary Materials. Additional data related to this paper may be requested from the authors.
