# Supplementary material for: Fabric muscle with a cooling acceleration structure for upper limb assistance soft exosuits
Source: Sci Rep. 2022 Jul 6;12:11398. doi: 10.1038/s41598-022-15682-w (PMC9259748; doi:10.1038/s41598-022-15682-w)
Supplement: Supplementary file 13 — Supplementary Legends. [file 41598_2022_15682_MOESM13_ESM.docx]

Supplementary video legends:

Video S1. Comparison of relaxation rates of fabric muscle during Joule heating and cooling under different cooling conditions.

Video S2. Comparison of flexion and extension responses under three different conditions.

Video S3. Repeating flexion-extension motion 10 times in condition 3.

Video S4. Example that the exosuit could assist both in lifting the load and in holding the lifting state

Video S5. Example of strength-assisted task performed by person wearing exosuit applying fabric muscle.

Supplementary data legends:

Data S1. Data of Figure 2 (c) and (e).

Data S2. Data of Figure 4 (a) and (b).

Data S3. Data of Figure 5 (c), (d), and (e).

Data S4. Data of Figure 6 (c) and (d).

Data S5. Data of Figure 7 (a), (b), and (c).

Data S6. Data of Figure 8 (b).

Data S7. Data of Figure 9 (a) and (b).
